# Optimization of Vertical Fixed-Bed Pyrolysis for Enhanced Biochar Production from Diverse Agricultural Residues

**DOI:** 10.3390/ma17123030

**Published:** 2024-06-20

**Authors:** Tasi-Jung Jiang, Hervan Marion Morgan, Wen-Tien Tsai

**Affiliations:** 1 Graduate Institute of Bio Resources, National Pingtung University of Science and Technology, Neipu Township, Pingtung 912, Taiwan; wttsai@mail.npust.edu.tw; 2 Department of Tropical Agriculture and International Cooperation, National Pingtung University of Science and Technology, Neipu Township, Pingtung 912, Taiwan

**Keywords:** biochar, thermal conversion, agricultural residues, environmental sustainability, environmental management

## Abstract

This study examines the pyrolysis of agricultural residues, namely, coconut shells, rice husks, and cattle manure, in a vertical fixed-bed reactor at varying temperatures from 300 to 800 degrees Celsius for biochar production. The research aimed to evaluate the potential of biochar as biofuels, adsorbents, and soil amendments. Proximate, ultimate, and elemental analyses were conducted to determine their composition and caloric values. Several analytical techniques were used in the physical and chemical characterization of the biochar (SEM, FTIR, BET). The results indicated that the highest S_BET_ values were achieved under different conditions for each biochar: 89.58 m^2^/g for BC-CS-700, 202.39 m^2^/g for BC-RH-600, and 42.45 m^2^/g for BC-CD-800. Additionally, all three biochars exhibited the highest caloric values at 600 °C. The results showed that 600 °C is the general optimal temperature to produce biochar from an assortment of biomass materials, considering their use for a variety of purposes. BC-CS-800 had the highest elemental carbon content at 93%, accompanied by a relative decrease in oxygen content. The van Krevelen diagram of biochar products shows that biochars derived from coconut shells and rice husks are suitable for use as fuels. Furthermore, FTIR analysis revealed the presence of oxygen-containing functional groups on the biochar surface, enhancing their pollutant adsorption capabilities. This study provides valuable insights into the scalable and environmentally sustainable production of biochar, emphasizing its role in improving soil quality, increasing energy density, and supporting sustainable agricultural practices.

## 1. Introduction

The exponential growth in the global population, coupled with the corresponding surge in food demand, poses substantial challenges to agricultural productivity [1]. Concurrently, agricultural practices are significant contributors to greenhouse gas emissions and environmental degradation [2]. Addressing these systemic issues, which include increased CO_2_ emissions [3], declining soil carbon levels [4], and global climate change [1], requires focused research on the development of biomass energy technologies and the enhancement of carbon sequestration methods in agricultural soils [5,6]. Among these, modern biomass pyrolysis, utilized in the production of biofuels and biochar, stands out as one of the most promising methods for achieving global-scale carbon capture and storage, offering a promising avenue for mitigating environmental impacts while enhancing agricultural sustainability [7,8].

Common agricultural residues such as rice husk [9], straw [10], corn cobs [11], sugarcane bagasse [12], coconut shell [13], and animal manure [6] make a significant negative contribution to the environment due to their vast availability and improper disposal methods. Traditionally viewed as waste, these residues not only contribute to pollution but also represent a lost opportunity for sustainable resource management [1]. However, these waste materials hold significant potential for sustainable use through their conversion into biochar [6]. Biochar is characterized by a stable carbon structure, high surface area, and large pore volume; these attributes have contributed to the versatility of its uses. [14,15]. This potential has been recognized globally, as studies have shown the feasibility of biochar produced from animal manure and plant residues in adsorbing pesticides [16], pharmaceuticals [17], hormones [18], and potentially toxic metals. In the context of global biochar production, the United States and Canada emerge as leading contributors within North America, driven by extensive agricultural applications and substantial investments in biochar technology. Europe also exhibits significant biochar production, with Germany, the United Kingdom, and Sweden being prominent players. Notably, Asia, with major contributions from China and Japan, is experiencing rapid growth in biochar production. This surge is largely attributed to expanding agricultural applications and an increasing focus on soil remediation [19].

Over the years, various technologies have been used in the production of biochar. These have been classified into three major techniques, with each category having sub-techniques. The first two are traditional technology (which includes the brick kiln and open hearth) and modern technology (such as pyrolysis, vacuum pyrolysis, microwave pyrolysis, gasification, and hydrothermal liquefaction), and the third is innovative technology (such as plasma- and electro-modified biochar). All of the mentioned technologies have been reported to have advantages and disadvantages with regard to production cost, time, required energy, and product yield. Among these technologies, the pyrolysis technique has received lots of attention because it is able to balance its pros and cons with regard to the mentioned advantages and disadvantages. 

The pyrolysis process is classified into three categories: slow, fast, and flash pyrolysis; each is optimized to enhance either the biochar, bio-oil, or syngas yield [20]. Slow pyrolysis is characterized by heating rates typically between 1 and 10 °C/min, conducted within a temperature range of 300–700 °C, strategically aimed at maximizing biochar production [21]. Fast pyrolysis focuses on converting solid biomass into liquid bio-oil, which has significant potential for energy applications. Operating within a temperature range of 300–700 °C and heating rates of 10–200 °C/s, fast pyrolysis decomposes biomass into vapors, aerosols, and some charcoal. Flash pyrolysis, the third form mentioned before, is used to produce syngas at higher temperatures of 900–1200 °C; it is characterized by short residence times and rapidly vaporizes the material into gaseous form [22]. The design of pyrolysis reactors is essential for the effective thermal decomposition of biomass, taking into account factors such as heating temperature, pressure, and vapor residence time. These factors are vital for converting biomass into various forms of energy, including gas, liquid, and solid fuels [23,24]. Among the various reactor types, the vertical fixed-bed reactor is noted for its simplicity and efficiency, particularly suited for low-throughput applications. This can be compared to other more complex reactor types, such as rotary kilns, which excel in processing diverse raw materials due to their uniform heat distribution, or microwave-assisted reactors, which have precise temperature controls [24].

In this research, the effects of operating a vertical fixed-bed reactor within a temperature range of 300 to 800 °C are evaluated. The effects that these conditions have on the biochar’s yield and quality are discussed in this article. This temperature range was specifically selected for its potential to significantly enhance the quality of biochar. The study thoroughly investigates the impact of different temperature settings on the biochar’s adsorption capacity, pore structure, and chemical stability. These factors are crucial for determining the efficacy of biochar as a versatile adsorbent, a soil amendment, and a biofuel. The aim is to establish a comprehensive understanding of the optimal conditions for biochar production that could maximize its applications in environmental management and sustainable energy practices. By bridging theoretical research and practical implementation, this study contributes valuable insights into scalable and environmentally sustainable biochar production techniques.

## 2. Materials and Methods

### 2.1. Materials

Raw biomass materials, namely, coconut shells, rice husk, and cow dung, were obtained from local agricultural cooperatives (Pingtung, Taiwan). These materials were then cleaned to remove any contaminants, followed by initial sun drying for about 48 h to remove moisture and save energy. After sun drying, they were placed in an oven at 105 °C to complete the drying process and further remove any trapped moisture. After complete drying, they were shredded and sieved to achieve uniform particle sizes between 1.410 mm (12-mesh) and 0.841 mm (20-mesh). The proximate analysis of each material was conducted, adhering to the standardized test methods established by the American Society for Testing and Materials (ASTM D-3172 [25]), to ascertain their contents of volatile matter, ash, and fixed carbon on a dry weight basis. Fixed carbon content was determined by the difference method using the formula FC = (100% − VM-Ash). Approximately 1 g of each dried biomass material was loaded into separate crucibles for each analysis. To ensure statistical significance, each material analysis was conducted in triplicate.

### 2.2. Thermochemical Characteristics Analyses of Agricultural Waste

#### 2.2.1. Thermal Decomposition Behavior—TGA-DTG

Thermogravimetric analysis was conducted using a TGA-51 instrument from Shimadzu Corporation Tokyo, Japan. The experimental protocol used a heating rate of 10 °C/min, chosen because previous data indicate that it falls within the slow pyrolysis range and yields favorable results [26]. These profiles are essential to understanding the pyrolytic behavior of the materials, a critical factor in optimizing the processing conditions to enhance both the yield and quality of the resulting biochar.

#### 2.2.2. Ultimate (Elemental) Analysis

Elemental analysis of the biochar samples was performed using the dry combustion method, where accurately weighed samples (~5 mg) were completely combusted at high temperatures. The resulting gaseous products were subsequently separated and quantified, comparing their compositions to standard samples of known composition. This analysis was conducted using the vario EL cube analyzer from Elementar GmbH, Langenselbold, Germany. The quantification process was repeated to confirm the consistency of the results. The contents of carbon, hydrogen, nitrogen, and sulfur in the biochar were determined and expressed as percentages of the total mass. The oxygen content was calculated by the difference method using the formula (O = 100%–C–H–N–S) to provide a complete profile of the elemental composition of the biochar. 

#### 2.2.3. Higher Heating Value (HHV) Analysis

The HHV or calorific values of the dried samples were measured using an adiabatic bomb calorimeter (Model No.: CALORIMETER ASSY 6200; Parr Co., Moline, IL, USA), which was ignited under an oxygen-rich atmosphere. Using about 0.5 g of the sample, each agricultural waste analysis was performed in duplicate based on the isoperibolic mode at 25 °C.

### 2.3. Carbonization Experiments

The primary equipment employed for the carbonization process was a vertical furnace with an 80 cm long tube and a 10 cm inner diameter. Nitrogen was selected as the protective gas during the process to prevent material oxidation at high temperatures. The carbonization experiments were conducted using a nitrogen flow of 500 cm^3^/min, a heating rate of approximately 10 °C/min, and a temperature range of 300 °C to 800 °C. The carbonization duration was set to 0 min, indicating that heating was terminated immediately upon reaching the preset temperature. The biochar yield was determined by comparing the mass of biochar obtained in each experiment to the initial mass of agricultural waste material using the following formula: Yield (wt%) = (biochar mass/initial biomass mass) × 100%. Approximately 5 g of raw material was introduced for each experimental run. To ensure consistency in experimental results, experiments under identical conditions were repeated. All experimental results were coded and recorded in the format “BC/Material-Temperature”; for instance, “BC/RH-700” represents the biochar product obtained from rice husk (RH) under a 700 °C condition for 0 min. This coding system facilitates the tracking of specific parameters for each experiment and provides a reliable basis for further analysis.

### 2.4. Porosity Analysis

The porosity characteristics of the obtained biochar, including the surface area, were assessed. Nitrogen adsorption–desorption isotherms measured at 77K (−196 °C) were employed for this purpose using the Accelerated Surface Area and Porosity Analyzer (ASAP 2020) from Micromeritics Co., Norcross, GA, USA. Prior to analysis, samples were degassed under vacuum at 250 °C to remove moisture and impurities. Pore characteristics were calculated based on relevant equations and models. The surface area was determined using the Brunauer–Emmett–Teller (BET) equation within a relative pressure range of 0.05 to 0.35.

### 2.5. Surface and Elemental Characterization of Biochar Products

#### 2.5.1. Scanning Electron Microscopy (SEM) with Energy-Dispersive X-ray Spectroscopy (EDS)

To visualize the surface morphology and elemental composition of the obtained biochar products, a scanning electron microscope (SEM) (model S-3000 N from Hitachi Co., Tokyo, Japan) equipped with energy-dispersive X-ray spectroscopy (EDS, 7021-H; HORIBA Co., Kyoto, Japan) was employed. The accelerating voltage was set at 15.0 kV. The EDS analysis results were compared and contrasted with the elemental analysis (EA) data [23].

#### 2.5.2. Fourier Transform Infrared (FTIR) Spectroscopy

Fourier transform infrared (FTIR) spectroscopy was also employed to analyze the surface chemistry of the biochar materials. FTIR spectra of the biochar sample surfaces were collected using an FT/IR-4600 spectrometer from JASCO Co., Tokyo, Japan. Prior to FTIR analysis, the samples were ground and mixed with Kbr (infrared grade) powder to a mixture containing approximately 1 wt% biochar. The mixture was then pressed into pellets with a diameter of 1.2 cm using a manual hydraulic press. Finally, transmission FTIR spectra were measured within a wavenumber range of 4000 to 400 cm^−1^ at a resolution of 4 cm^−1^ [27].

## 3. Results

### 3.1. Properties of CS, RH, and CD Feedstocks

Table 1 presents an in-depth analysis of the thermochemical properties of coconut shell (CS), rice husk (RH), and cow dung (CD) as agricultural residues for biochar production. CS is notable for its high fixed carbon content of 17.97%, signifying exceptional stability and carbon retention, thereby reinforcing its applicability in biochar applications targeted at carbon sequestration. The possible uses of CS biochar may be quite different from those of RH and CD due to their lower fixed carbon contents of 10.69% and 6.31%, respectively [27]. Coupled with its high calorific value of 20.92 MJ/kg, CS is recognized as a beneficial resource for both soil amendment and bioenergy production [28]. 

When observing the volatile matter content of the biomass samples, it was noted that CD had a volatile matter content of 83.02%, while CS’s VM content was recorded at 81.58%, and RH’s was lower at 68.59%. The presence of high VM content may potentially result in increased gaseous emissions during pyrolysis [23]. The effective management of these emissions is essential for exploiting the energy potential while mitigating environmental impacts. Furthermore, the minimal ash content in CS (0.45 ± 0.12%) is associated with enhanced biochar quality and a higher calorific value, providing substantial advantages for energy production. When comparing this result with those of CD and RH, it was observed that the ash content of these biomass materials was higher than that of CS. RH’s ash content was recorded at 13.38%, which is similar to the range recorded in the previous literature, 10–12% [29], and that of CD was recorded at 6.65%, which falls into the 5–7% range from the literature [30]. Such ash content, indicative of the residual inorganic minerals post-combustion, plays a critical role in energy utilization due to the presence of alkali metals such as silicon [27,30]. This variability could potentially complicate energy utilization, raising concerns about slagging and fouling in industrial boilers or gasifiers [27].

The elemental analysis, as observed in Table 1, shows the organic content. As expected for biomass materials, carbon and oxygen constituted the largest proportions of the materials. Among the three biomass samples, CS had the highest carbon percentage, recorded at 50.92%, while cow dung and rice husk were around 47% and 43%, respectively. The presence of carbon in biomass is highly related to the structures of the macromolecules that biomass materials are composed of. Rice husk had the highest oxygen content at 49.83%, while cow dung and coconut shell were about 44% and 41%, respectively. The carbon content and oxygen and hydrogen contents, along with the respective O/C and H/C ratios, are important pieces of information, especially if the intended purpose of the material is for dry fuel use [31]. The other recorded elements in the analysis were nitrogen and sulfur. Both of these elements made up less than 1.5% of all biomass samples. 

**Figure 1:**
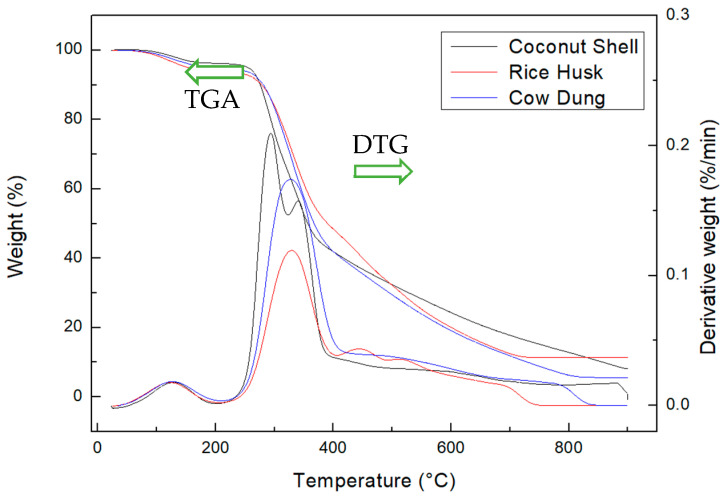
TGA and DTG curves of the three biomass samples (CS, RH, and CD) recorded at a constant rate of 10 °C/min.

Figure 1 presents the thermogravimetric analysis (TGA) and differential thermogravimetric analysis (DTG) results for three biomass samples: coconut shell, rice husk, and cow dung. TGA studies the degradation of biomass with respect to changes in temperature. A slight decrease observed around 100 °C is due to the volatilization of residual moisture trapped inside the biomass, even after drying [32]. All three samples show significant degradation starting at around 250 °C, primarily due to hemicellulose decomposition. As the temperature increased, the degradation rates varied: coconut shell showed the highest DTG peak at 0.2%/min, indicating rapid biomass degradation, whereas cow dung and rice husk exhibited lower peaks at 0.15%/min and 0.1%/min, respectively, indicating more stable degradation. Most of the weight loss occurred between 250 and 350 °C, suggesting that hemicellulose was the easiest component to degrade [33]. The coconut shell’s DTG curve showed two peaks at around 275 °C and 365 °C, indicating non-continuous thermal degradation due to its complex fiber network and high density. Similar patterns are observed in the degradation of other hardwoods [34,35]. The residual matter at the end of the experiment was highest for coconut shell at 20%, followed by rice husk at 15% and cow dung at 10%. The difference in residual matter is an indication of the initial strength and composition of the biomass. In particular, those with higher lignin content result in greater residual matter, as shown in the case of coconut shells [36]. According to the literature, hemicellulose degrades first within the temperature range of 200–380 °C due to its chemical structure consisting of pentoses and hexoses [37]. Cellulose decomposes between 250 and 380 °C, being primarily composed of glucose units, making it stronger than hemicellulose but weaker than lignin, which provides plant flexibility [38,39]. Lignin, responsible for plant rigidity, decomposes between 600 and 900 °C due to its complex phenolic macromolecular structure [40,41]. The TGA and DTG curves seen in Figure 1 follow the general degradation patterns of these three components, indicating that the biomass samples were composed of all three biomolecules [42].

### 3.2. Mass Yields and Calorific Values of Biochar Products

#### Mass Yields and Calorific Values of Biochar Products

The thermochemical conversion of agricultural residues into biochar was examined across a range of pyrolytic temperatures (300 °C to 800 °C), focusing on the yield, surface area, calorific value, and physicochemical properties. The yield of biochar inversely correlated with the pyrolysis temperature [42,43], exhibiting a decrease from 57.63% to 21.80% for BC/CS, 61.63% to 36.82% for BC/RH, and 68.99% to 30.22% for BC/CD as the temperature increased. The decrease in biomass yields at elevated temperatures can be attributed to the degradation of biomass macromolecules. With the rising temperature, these molecules can degrade and vaporize, leaving behind a stronger carbon matrix [44]. On the other hand, the pyrolysis temperature tends to have a different effect on the specific surface area of biochar materials. There were noticeable differences in the results obtained from this analysis. Firstly, it is agreed that an increase in pyrolysis temperature will also cause an increase in the surface area; however, in some cases, the trend may not be continuous because of the type of biomass feedstock. This was the case for the three biomass feedstocks. The highest S_BET_ for CS was recorded at 700 °C with a value of 89.58 m^2^/g; before that, there was a significant change in S_BET_ to 84.28 m^2^/g at 600 °C. Lower temperatures between 300 and 500 °C played an insignificant role in opening the pores of the CS material. It is noticeable, though, that there was a slight decrease to 81.56 m^2^/g in the S_BET_ of the CS as the pyrolysis temperature was further increased to 800 °C. As explained in the literature [45], this decrease may have been the result of the clogging of pores due to the incomplete vaporization of compounds in the carbon matrix. The increase in temperature from 700 °C to 800 °C could have initialized a new sequence of degradation that was unable to finish at 800 °C, hence leaving residues inside the biochar pores. This makes it possible to agree with the literature that the residence time during pyrolysis is just as important as the maximum pyrolysis temperature [14]. RH biochar followed a similar trend as well, with its highest S_BET_ of 202.39 m^2^/g recorded at 600 °C, after which the value started to decrease as the temperature further increased, eventually resulting in a low S_BET_ of 64.34 m^2^/g at 800 °C. The initial biomass composition may have played a significant role in the decrease in S_BET_ with regard to a rise in temperature. The third biomass that was observed was CD; the highest S_BET_ for this material was recorded at the highest temperature, 42.45 m^2^/g at 800 °C. However, these are relatively low surface areas, which is attributed to the properties of the initial biomass. From the S_BET_ results, it can be inferred that the residence time at a desired temperature is just as important as the maximum temperature if the increase in the specific surface area is a desired characteristic [27,28]. A notable increase in calorific value was consistently observed across all three residues as the temperature approached 600℃. At this pivotal thermal threshold, CS biochar exhibited a significant calorific value of 37.61 MJ/kg, while BC/CD and BC/RH were measured at 22.36 MJ/kg and 25.31 MJ/kg, respectively. Relative to the inherent calorific values of the raw materials, as delineated in Table 2, the enhancement factors stood at an impressive 180% for CS, 153% for RH, and 120% for CD. These findings are integral to understanding the thermochemical conversion efficiency and optimizing pyrolysis parameters for each type of biomass. The sharp increase in calorific value aligns with a probable reduction in oxygen content during pyrolysis, indicative of a higher carbon concentration in the charred product [29]. This trend is critical for applications where biochar is envisioned as a renewable energy source due to its improved energy density.

### 3.3. Physical Characteristics of Biochars

**Figure 2:**
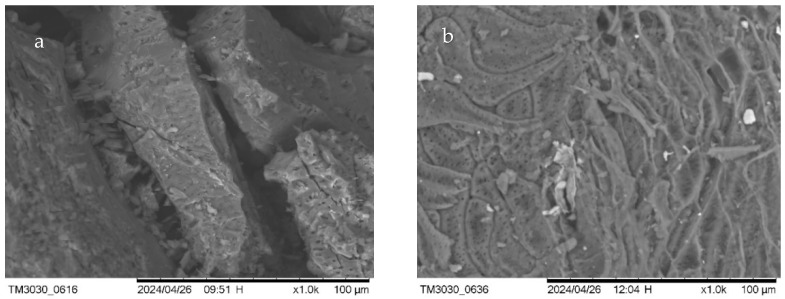
SEM images of CS biochar. (**a**) BC/CS-300 (left); (**b**) BC/CS-800-0 (right).

**Figure 3:**
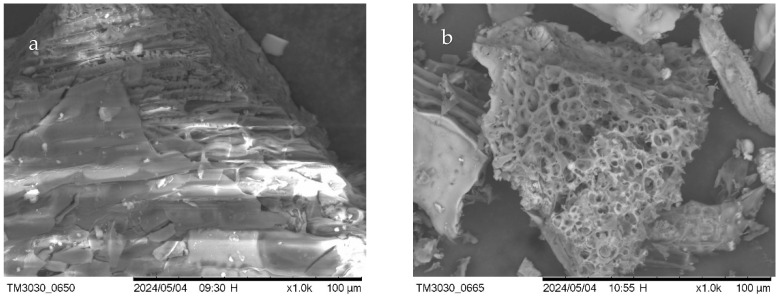
SEM images of RH biochar. (**a**) BC/RH-300 (left); (**b**) BC/RH-800-0 (right).

**Figure 4:**
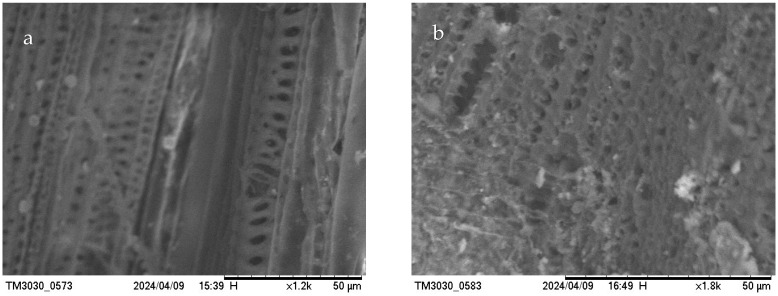
SEM images of CD biochar. (**a**) BC/CD-300 (left); (**b**) BC/CD-800-0 (right).

In order to observe the porous texture of the resulting biochar, BC/CS-300 & 800 (Figure 2), BC/RH-300 & 800 (Figure 3), and BC/CD-300 & 800 (Figure 4) were examined using scanning electron microscopy (SEM) and energy-dispersive X-ray spectroscopy (EDS) (Table 3). SEM imaging confirmed the development of an extensively porous structure with increased pyrolysis temperature, indicative of an augmented surface area that facilitates a higher adsorption capacity. Complementarily, EDS spectra revealed that the BC/CS-800 sample was predominantly carbonaceous, boasting an 85.519% carbon content, aligning with its dense and homogeneous surface morphology observed in SEM.

Careful observation of the EDS data presented in Table 3 shows that the carbon contents of BC/RH-800 and BC/CD-800 were lower, recorded at 68.94% and 75.17%, respectively. These lower carbon contents compared to that of BC/CS-800 corroborate the results of the calorific values discussed previously, as well as the initial biomass composition. The oxygen content recorded is also aligned with this result. Toscano and Pedretti (2009) found a direct relationship between the carbon content of biochar and its calorific value, as well as the elemental composition of the initial biomass [46]. Spokas (2010) further supported this correlation, noting that the presence of higher oxygen content inversely affects the carbon content and calorific value of biochar [47]. The other elements that were recorded in the EDS spectra were silicon and phosphorus. Silicon had its highest percentage in BC/RH-800 at 6.69% and the second highest in BC/CD-800 at 2.95%. The presence of silicon at these percentages is consistent with the fact that the initial biomass was rich in this mineral source. Another reason why silicon is found in biochar produced at higher temperatures, particularly those above 650 °C, is that the silicates need higher temperatures to crystallize in the biochar matrix [48]. The lower levels of phosphorus recorded in BC/CD-800 (1.04%) may have been a result of the initial biomass; the degradation of the biomass material may have led to the formation of phosphorus-containing compounds on the biochar’s surface. Phosphorus transformation during pyrolysis shows that more stable phosphorus species are formed at higher pyrolysis temperatures, which may explain the lower availability of phosphorus in biochar produced at these temperatures [27,49,50]. The presence of these elements, coupled with a more heterogeneous and fibrous morphology, may enhance the nutrient-rich profile of the biochar, suggesting its suitability for soil amendment applications [51]. The elemental compositions, not summing to 100%, point toward the complexity of biochar matrices, incorporating various macro- and micro-nutrients beneficial for environmental remediation. The combined SEM-EDS analysis thus provided a multifaceted characterization of biochars, underscoring the influence of pyrolysis on physicochemical properties that are pivotal for their tailored application in sustainable agriculture and environmental management.

### 3.4. Chemical Characteristics of Biochars

**Figure 5:**
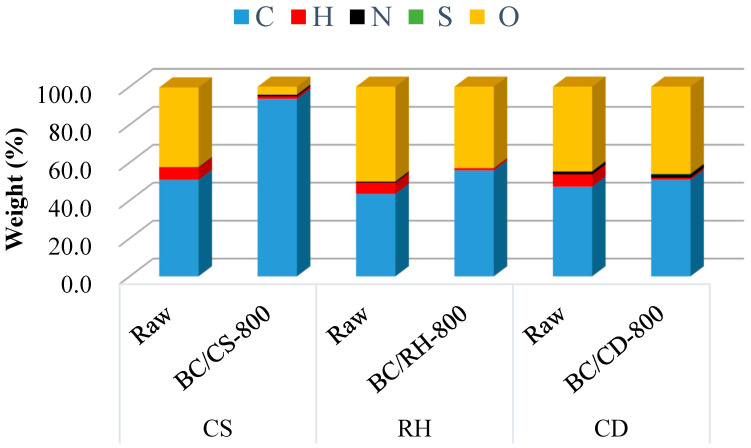
Ultimate analysis of the raw biomass and biochars (800 °C).

**Figure 6:**
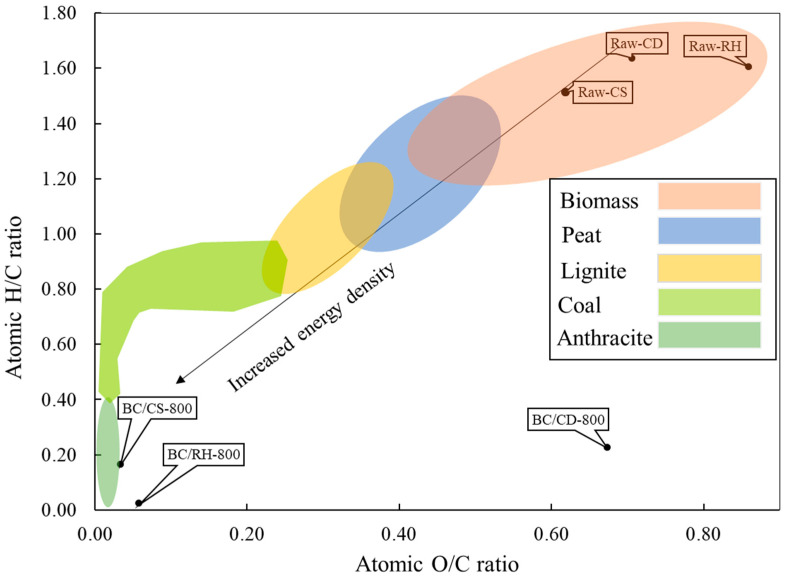
The van Krevelen diagram of the raw biomass and biochar (800 °C).

Elemental analysis was performed on the raw biomass and the corresponding biochar from BC/CS, BC/RH, and BC/CD (Figure 5 and Figure 6). Only the analysis of biochar produced at 800 °C is used in the comparison to simplify the explanation of the data. There was a general increase in carbon content with rising temperatures, although losses in oxygen, hydrogen, and sulfur were recorded. The results indicated that the resulting biochar had higher carbon contents, ranging between 51% and 93%, compared to the carbon content of less than 50% for the raw biomass materials. This enrichment of carbon content highlights the thermochemical transformation inherent to the pyrolysis process, thereby enhancing the potential of biochar as a renewable energy source [21,23]. Changes in the elemental composition of biochar are particularly noteworthy, for example, the absence of nitrogen in the RH biochar samples. On the other hand, post-pyrolysis, BC/CD-800 showed a slight increase in nitrogen content from 1.44 to 1.78%. Despite numerous authors reporting a decrease in nitrogen content in biochar with pyrolysis temperature [52], this study observed a relative increase in nitrogen content within the biochar samples with rising temperature, which can be explained by the incorporation of nitrogen into thermally stable and non-volatile complex structures [53].

The reduction in the O/C ratio indicated improved fuel properties, particularly that observed in the RH biochar (Figure 6), signifying an approximate 60% decrease and, hence, an improved energy profile. However, the O/C ratio in CD biochar increased, potentially indicating a lower calorific value. These changes in chemical structure through the pyrolysis of some biochar samples not only enhance the candidacy of biochar for bioenergy applications but also comply with the standards set by the European Biochar Certificate (EBC), which specifies maximum molar ratios of O/C and H/C for quality assurance and environmental sustainability. The EBC mandates that the molar ratios of O/C and H/C be less than 0.4 and 0.7, respectively [54].

#### FTIR Characterization

**Figure 7:**
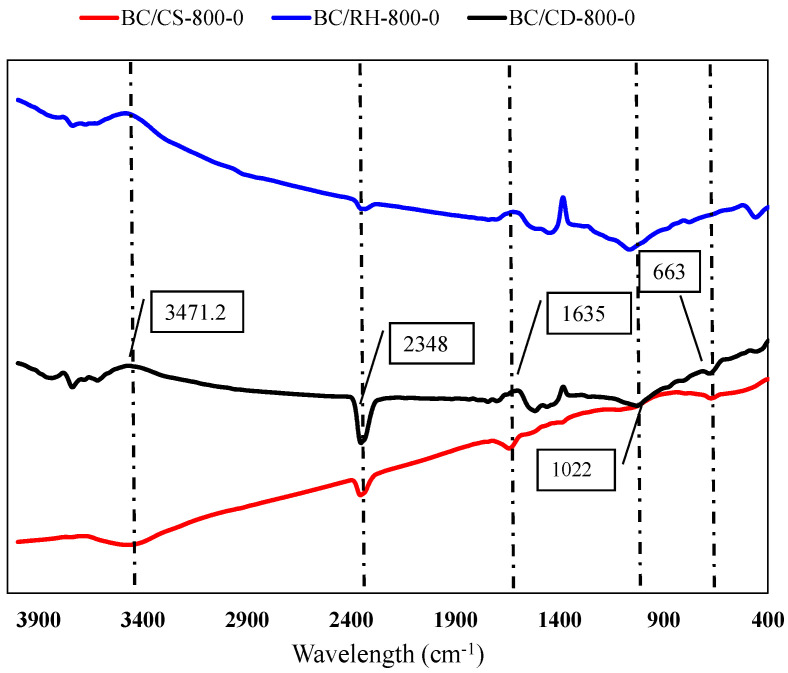
FTIR spectra of the resulting biochar (800 °C).

The FTIR spectrum shown in Figure 7 explains the results of the biochar produced at 800 °C with no retention time. The results of the spectral analysis are used to identify the functional groups present on the biochar surface, which play an instrumental role in determining the resulting potential biochar applications. Careful analysis of the spectrum indicates the presence of a peak at wavenumber 3471 cm^−1^. The presence of this stretch confirms the biochar’s hydrophilicity, indicative of adsorbed water molecules and the presence of the hydroxyl group (O-H) [55]. The absorbance at around 2348 cm^−1^ aligns with the presence of aromatic and aliphatic structures, resonating with C–H and C–C bonds, as documented in the literature [27,56]. The biochar’s potential to enhance sorption capacities for heavy metals in soil can be partly attributed to these structures, thanks to the electrostatic attraction facilitated by oxygen-containing functional groups [42]. In the mid-infrared region, the band between 1550 and 1710 cm^−1^ is typically associated with C=O stretching in carbonyl groups or C=C stretching in aromatic rings, reflecting the biochar’s complex aromatic carbon structures and inorganic mineral contents. The sharp peaks observed in the range of 966–1079 cm^−1^ may denote C-H-group deformations. These FTIR spectral features not only elucidate the chemical composition changes incurred during the pyrolysis of BC/CS-800, BC/RH-800, and BC/CD-800 but also highlight the functionality of biochar in environmental contexts, enhancing our understanding of how biochar can be used in a variety of applications, such as soil amendment [57], heavy metal remediation [58], water filtration [59], or solid fuel [15].

## 4. Discussion

In this research, three agricultural residues were investigated: coconut shell, rice husk, and cattle manure. These represent the wide variety of sources that contribute to agricultural residues. In the reviewed literature, there have been several suggestions on how agricultural residues can be valorized into biochar and be used to contribute to environmental development. Examples of these suggestions are those of Lehmann and Joseph [60], who suggested four motivational objectives for biochar application, i.e., soil improvement, waste management, climate change mitigation, and energy. These objectives can be used individually or in combination with each other. However, concerning the results of our investigation and the quality of the biochar produced in this research, it can be deduced that these biochars would be best employed in simpler applications, such as improving soil quality and energy sources. Below, these ideas will be discussed further. 

### 4.1. Impact of Biochar Uses for Soil Quality 

The application of biochar can improve soil health through enhanced nutrient retention, microbial activity, and soil structure. Coconut shell biochar, with a fixed carbon content of 17.97% and a specific surface area of 89.58 m^2^/g at 600 °C, would be particularly effective in boosting soil biological activity and immobilizing heavy metals [61]. Rice husk biochar, with a fixed carbon content of 10.69% and specific surface area of 202.39 m^2^/g at 600 °C, can significantly reduce nitrogen leaching, enhance soil pH, and increase the cation exchange capacity (CEC), fostering a healthier soil environment [62]. The third biochar was cow dung: though lower in fixed carbon content at 6.31%, it would provide substantial improvements in soil fertility and structure because of its specific surface area of 42.45 m^2^/g at 800 °C. Collectively, these biochars can enhance soil quality by improving nutrient availability, increasing microbial diversity, and enhancing water retention.

There is a need for further research that can focus on investigating the long-term effects of biochar application on soil health, including nutrient cycling, microbial diversity, and overall soil fertility [63,64]. For example, biochar amendments in sandy soils have been shown to reduce bulk density and increase water retention. Soils treated with various types of biochar (woodchip, waterweed, poultry litter, and bagasse) at different rates showed significant improvements in water retention capacity, with increases in readily available water of up to 191% at a 10% biochar content [65]. Additionally, refining pyrolysis conditions to maximize the yield and quality of biochar is essential [44]. This includes experimenting with different temperatures, residence times, and feedstock combinations to produce biochar with optimal properties for soil enhancement [27,28]. 

### 4.2. Impact of Biochar Uses for Energy

Biochar can also serve as a valuable renewable energy source. Coconut shell biochar achieved a high calorific value of 37.61 MJ/kg at 600 °C, making it an efficient and economically viable energy solution [10,27]. Rice husk biochar, with a calorific value of 25.31 MJ/kg at 600 °C, provides a suitable balance between a function in energy production and soil enhancement benefits, making it suitable for integrated sustainable development projects [62]. Thirdly, cow dung biochar, although lower in calorific value at 22.36 MJ/kg at 600 °C, would still be a suitable energy source; this is because even the raw dried biomass is used in developing nations as a fuel source for cooking.

In order to explore the extensive applications of biochar in terms of energy uses, comprehensive economic analyses should be conducted to determine the cost-effectiveness of biochar production and application at different scales, from small farms to large agricultural operations [66]. Research should also explore the feasibility of biochar production in various geographic regions and agricultural contexts [67,68]. Finally, the applications and uses of biochar with regard to soil or energy should be accompanied by a feasibility study that can make the technology available to as many persons as possible while maintaining the spirit of sustainability.

## 5. Conclusions

This study assessed the transformation of coconut shells, rice husks, and cattle manure into biochar via pyrolysis, analyzing their evolving physical and chemical properties across a temperature range of 300 to 800 degrees Celsius. The research outcomes indicated that coconut shell biochar exhibited exceptional characteristics for bioenergy utilization and environmental applications due to its high carbon content and thermal stability, making it particularly suitable for soil carbon sequestration and as a renewable biofuel. Coconut shell biochar showed a significant increase in calorific value, peaking at 37.61 MJ/kg at 600 °C, illustrating its potential as an alternative energy source. In contrast, rice husk biochar, with its high mineral content and optimal O/C ratio, is ideally suited for soil amendment applications, enhancing soil fertility and structural properties, demonstrated by its dramatic increase in surface area to 202.39 m^2^/g at 600 °C. Furthermore, cattle manure biochar, enriched with nitrogen, holds the potential to significantly boost soil fertility, especially in agricultural ecosystems. Physical characterizations through SEM revealed that the biochars developed extensive porous structures at elevated temperatures, enhancing their adsorption capacities—a vital feature for environmental remediation. Chemical analyses showed that the biochars’ carbon contents increased with temperature, while reductions in oxygen, hydrogen, and sulfur contents improved their energy profiles. In general, it was observed that 600 °C is a suitable temperature that can produce biochar from a diverse source of materials that can be used in a variety of applications, such as soil amendment, absorbent, and nutrient remediation.

## Figures and Tables

**Table 1:** Proximate and elemental analyses of the biomass samples.

Thermochemical Property ^a^	Value ^b^		
	Coconut Shell (CS)	Rice Husk (RH)	Cow Dung (CD)
Proximate analysis (wt%) ^a^			
Volatile matter (wt%)	81.58 ± 304	68.59 ± 0.00	83.02 ± 0.00
Ash (wt%)	0.45 ± 0.12	13.38 ± 0.00	6.65 ± 0.00
Fixed carbon (wt%)	17.97	10.69	6.31
Elemental analysis (wt%) ^a^			
Carbon (C)	50.92 ± 0.98	43.51 ± 1.39	47.31 ± 0.57
Hydrogen (H)	6.43 ± 0.1	5.82 ± 0.20	6.45 ± 0.08
Oxygen (O)	41.98 ± 0.46	49.83 ± 1.82	44.52 ± 0.64
Nitrogen (N)	0.18 ± 0.10	0.56 ± 0.10	1.44 ± 0.00
Sulfur (S)	0.09 ± 0.11	0.28 ± 0.12	0.29 ± 0.00
Calorific value (MJ/kg)	20.92 ± 0.07	16.59 ± 0.14	18.58 ± 0.05

^a^ On a dry basis; ^b^ average value of two/three determinations.

**Table 2:** Characterization of biochar produced from agricultural residues at varied pyrolysis temperatures.

Biochar Sample ^a^	Temperature (°C)	Yield (%) ^b^	S_BET_ ^c^	Calorific Value (MJ/kg)
BC/CS	BC/CS-300	57.63	2.51	26.36
	BC/CS-400	32.35	0.72	33.77
	BC/CS-500	32.34	0.31	35.17
	BC/CS-600	27.75	84.28	37.61
	BC/CS-700	26.19	89.58	36.31
	BC/CS-800	21.80	81.56	37.12
Rice husk	BC/RH-300	61.63	0.21	22.34
	BC/RH-400	50.62	4.48	23.95
	BC/RH-500	46.14	18.53	24.46
	BC/RH-600	45.76	202.39	25.31
	BC/RH-700	39.19	125.76	24.68
	BC/RH-800	36.82	64.34	24.41
Cow dung	BC/CD-300	68.99	-- ^d^	20.74
	BC/CD-400	45.07	-- ^d^	22.01
	BC/CD-500	37.84	12.18	21.47
	BC/CD-600	37.57	6.71	22.36
	BC/CD-700	35.43	8.19	21.79
	BC/CD-800	30.22	42.45	21.93

^a^ On a dry basis; ^b^ the mean ± standard deviation of three determinations using the same sample solution; ^c^ BET surface area; ^d^ the data cannot be measured.

**Table 3:** Elemental contents of the resulting biochar products determined from EDS spectra.

Elemental Content (wt%)	BC/CS-800	BC/RH-800	BC/CD-800
Carbon (C)	85.52	68.94	75.17
Oxygen (O)	14.48	24.26	19.00
Silicon (Si)	--	6.69	2.95
Phosphorus (P)	--	--	1.04

Note: The sums of the percentages do not reach 100% due to the presence of other trace elements not included in the table.

## Data Availability

The original contributions presented in the study are included in the article, further inquiries can be directed to the corresponding authors.
